# Supplementary material for: The psychosocial determinants of adherence to home-based rehabilitation strategies in parents of children with cerebral palsy: A systematic review
Source: PLoS One. 2024 Jun 12;19(6):e0305432. doi: 10.1371/journal.pone.0305432 (PMC11168686; doi:10.1371/journal.pone.0305432)
Supplement: Attachment — Submitted filename: Rebuttal letter April 10, 2024.doc [file pone.0305432.s002.doc]

**Rebuttal letter**

We wish to express our sincere appreciation to the editor and reviewer for their insightful comments. We have done our best to address all the issues raised and we hope that the final version of this manuscript is much improved due to their comments.

**Note:** This table corresponds to the manuscript with track changes. It means that page numbers correspond to the manuscript with track changes.

| **N** | **Reviewer 1** | **Reviewer’s comments** | **Page and line on which addressed** | **How comments were addressed** |
| --- | --- | --- | --- | --- |
|  | **General comments** | **Comments to the Author**  1. Is the manuscript technically sound, and do the data support the conclusions?  Reviewer #1: Yes  Reviewer #2: Yes  2. Has the statistical analysis been performed appropriately and rigorously?  Reviewer #1: N/A  Reviewer #2: Yes  3. Have the authors made all data underlying the findings in their manuscript fully available?  Reviewer #1: Yes  Reviewer #2: Yes  4. Is the manuscript presented in an intelligible fashion and written in standard English?  Reviewer #1: Yes  Reviewer #2: Yes | Whole document | We really appreciate the reviewer 1 and reviewer 2 for the overall appreciation of the quality of this manuscript. |
| 1 | Reviewer #1: | Reviewer #1: The paper presents a review of studies targeting home-based rehabilitation strategies in parents of children with cerebral palsy. The topic is interesting and might provide insights on how to increase adherence or adapt the program setting. | NA | **We appreciate the reviewer for the constructive comments:**  *We really appreciate the reviewer 1 for his flawless point of view on the theoretical and practical implication of the current systematic review within the filed.* |
|  |  | In addition, more details are required for some metrics and how they are measured, in particular, for metrics like self-efficacy or adherence. Please see my detailed comments below: | Page 9, line 1801, table 1. | ***We appreciate the reviewer for the constructive comment.***  *The more details regarding the term adherence were initially provided in S1 table especially in its column titled “Concept (and measure) of Adherence to HEP.” Due to the variations in measuring adherence across studies, we included in this review how each included study measured adherence. We revealed that measurement approaches ranged from self-designed questionnaires with a few questions to comprehensive, validated questionnaires with up to 28 items. Many studies utilized a 5-point Likert scale to measure adherence, capturing a range from "never" to "always" engaging in prescribed exercises. Additionally, qualitative methods such as semi-structured interviews and focus group discussions were employed to gather deeper insights into adherence challenges. This inclusion of both quantitative and qualitative measures highlights the complexity of assessing adherence and the diverse nature of home exercise programs. This refined overview is presented in the manuscript to better articulate the multifaceted approach to adherence measurement in pediatric rehabilitation contexts.*  *We are sorry that you didn’t you didn’t see this table but this we have submitted them inside the manuscript instead of being a supplement as we did before.* |
|  |  | In the abstract, you define the term GMF (gross motor function), however, in the conclusion of the abstract, you use the term FGM. Do both terms describe gross motor function, or does the latter define something different? | Page 1, line 44. | **Thanks for the comment. We apologize for this error which was corrected as follows:**  *Our findings highlight that factors influencing AHEP are multifactorial. Some of them are conditions that are difficult to change such as* ***GMF*** *or the economic and social conditions of the family.* |
|  |  | In the introduction, the first sentence starts with the term CP; however, this abbreviation is introduced in the following sentence. Please address this issue. | Page 3, Line 198 | **We apologize for this error that was corrected in the manuscript as follows:**  *Cerebral palsy (CP) is the largest diagnostic group treated in paediatric rehabilitation. ^[1,2]^ CP is a spectrum of motor development problems caused by persistent damage to the immature brain ^[3]^.* |
|  |  | 4. In the introduction, you mention that the prevalence of CP in some countries is "1.8-2.3% per 1000 children," or in other words, 0.18‰-0.23‰. In the following sentence, you write that in Africa, the respective rate is "2-10 per 1000 live births." Do you mean 2-10% per 1000 children or 2‰-10‰? | Page 3, line 209 | **We appreciate the reviewer for the constructive comments.** You’re right it’s 1.8‰-2.3‰ and 2‰-10‰. we have corrected in the manuscript as follows:  *According to data from national cerebral palsy registries and population-based research in Europe, Australia, and the United States, the prevalence of CP is around* ***1.8-2.3 per 1000*** *children ^[8–10]^. In contrast to the high-income countries, the prevalence of CP in Africa might reach up to* ***2-10 per 1000 children*** *^[11,12]^.* |
|  |  | C-CP is defined in the abstract. Please introduce this term in the main text as well. | Page 3, line 216 | **We appreciate the reviewer for the constructive comments: it was addressed in the main text as follow:**  *Early intervention allows children with cerebral palsy (C-CP) to reinforce their remaining potential abilities by tackling their initial movement limitations.* |
|  |  | Lines 90-91: Is this applicable only for families living in rural areas (e.g., involving parents in treatment decisions)? | Page 3, line 224 | **We appreciate the reviewer for the constructive comments, we have corrected in the manuscript as follows:**  *Furthermore, HEP is crucial for families as it saves time and money, involves parents in treatment decisions and allows them to choose the best options for their child.^[1,23]^* |
|  |  | Line 125: It is written that "The search terms were specially designed for the relevant database." Do you imply that the search terms were not the same for all the databases but differed across them? The terms mentioned in lines 126-131, are they connected with an AND or OR? Please provide a sample query you used for one database (e.g., PubMed). Did you also include terms starting with a prefix (e.g., adheren* which includes the terms adherence and adherent as keywords)? | Page 5, Line 795 | **We appreciate the reviewer for the insightful comment:**  *The search strategies for this review were established by applying Boolean operators ‘AND’ and ‘OR’ to effectively connect the descriptors, facilitating a focused yet exhaustive search. These descriptors were derived from keywords and MeSH terms search (in PubMed) in each database's metadata system. Therefore, the strategy was carefully developed for each database, considering its unique indexing system and search functionalities to guarantee the identification of the most pertinent and extensive collection of literature.*  *The search terms covered a spectrum of terms* *relating to home exercise programs,* *adherence,* *diverse influencing factors and cerebral palsy. To ensure the inclusion of a wide range of studies, the search incorporated terms with prefixes. The sample query for PubMed was constructed as follows: ("exercise" OR "therap*" OR "physiotherap*" OR "home* *physiotherap*" OR "home exercise*" OR "rehabilitation exercise*" OR "physical therapy") AND ("adheren*" OR "complian*" OR "involvement") AND ("psychosocial factor*" OR "psychological factors" OR "motivat*" OR "barrier*" OR "facilitat*") AND ("cerebral pals*" OR "neurodevelopmental disorder*" OR "pediatric neurology").* |
|  |  | Line 133: Please include a reference to the Covidence program. | Page 5, line 803 | **We appreciate the reviewer for the constructive comments:**  *We imported all references into our Covidence database* ***(***[***www.covidence.org***](http://www.covidence.org/)***)*** *for deduplication against other sources and screening according to the same inclusion criteria.* |
|  |  | Lines 135-138: Did you also include studies with computer-based rehabilitation for such children? | Page 5, line 810  Page 5, line 2466 | **Thank you for your query on the inclusion of computer-based rehabilitation studies. Our review focused on psychosocial determinants of parental adherence to home-based rehabilitation for children with cerebral palsy.** Upon review, we found that studies on computer-based rehabilitation did not provide the necessary insights into these psychosocial determinants. Thus, they were not included in our analysis. We revised in the method section and the discussion as follows:  *Studies were considered eligible for inclusion if they (i) involved parents of children with CP who were younger than 18 years; (ii) described exercises that were assisted by a caregiver rather than performed independently by the patient; and (iii) were published in English. Notably, studies that presented both clinic and home-based exercise adherence data were included, provided the home-based data were reported separately. Exclusions were made for (i) review articles; (ii) studies involving children without a confirmed CP diagnosis; (iii) studies not available in full text; and (iv)* ***computer-based rehabilitation studies due to a lack of reported data on psychosocial determinants of adherence from parents’ perspectives, which was a critical focus of our review.***  **Revision in Discussion, especially in subsection titled “implication for research.”**  *In terms of research, further studies are warranted to determine the nature of the relationships between HEPA and each of the five identified consistent predictors. It is crucial to understand whether certain groups of parents or specific circumstances amplify these predictors’ effects, and to what extent other factors may mediate or moderate the relationship. Coupled with the expanding research on behavior modification techniques ^[51]^, these findings may guide the development of highly effective strategies to boost HEPA. Identifying the most impactful adherence behavior factors allows for a targeted approach in selecting of behavior modification strategies for these interventions.* ***Furthermore, despite acknowledging computer-based rehabilitation's increasing role in managing cerebral palsy and its influence on adherence, there is a noticeable gap concerning its psychosocial dimensions. This observation underscores the necessity for research focused on the psychosocial aspects of adhering to such rehabilitation modalities.*** *Finally, future studies addressing the methodological limitations identified in this review, particularly the challenges in monitoring home-exercise adherence, is essential for enhancing the evidence base regarding HEPA predictors.* |
|  |  | Line 143: Repetition related to Covidence. | Page 5, line 791 | **Thank you for the constructive comments, the authors have revised the section.** This revision aims to streamline the description of the use of Covidence, clarify the selection process, and avoid the unnecessary repetition of the tool's mention, focusing instead on its role in the research process. The following correction was made in the manuscript:  *Two separate researchers independently conducted the search and evaluated studies for eligibility based on the inclusion criteria (JN, LU) using the Covidence database (*[*www.covidence.org*](http://www.covidence.org/)*). In instances of disagreement, a third reviewer (a team of MN, LC, and FC) was consulted to resolve the discrepancies. Studies were considered eligible for inclusion if they (i) involved parents of children with CP who were younger than 18 years; (ii) described exercises that were assisted by a caregiver rather than performed independently by the patient; and (iii) were published in English. Notably, studies that presented both clinic and home-based exercise adherence data were included, provided the home-based data were reported separately. Exclusions were made for (i) review articles; (ii) studies involving children without a confirmed CP diagnosis; and (iii) studies not available in full text.*  *The selection process began with the Boolean combination of descriptors, followed by a review of titles, abstracts, and finally, full texts. All references were imported into Covidence for deduplication and screened for eligibility based on the established criteria. The number of articles identified through electronic databases and the reasons for exclusion were rigorously documented in Figure 1. Following* *identification of relevant papers, their reference lists were manually searched for additional pertinent literature. At the conclusion of the selection phase, chosen papers chosen were organized for data extraction, tabulation, and analysis, and subsequent interpretation and reporting.*Top of FormBottom of Form |
|  |  | Table and figures mentioned in the text (e.g., Table 1, line 156) are not available in the document or the PLOS ONE review system. |  | It is surprising! We are sorry that you didn’t you see table and figures, which was initially submitted as supplement files, but now we submit it inside the manuscript to ensure that you see it. |
|  |  | Lines 196-197: No study was conducted between 2005 and 2013? | Page 9, line 1848,  Table1 | **We appreciate the reviewer for the constructive comment.**  You are right, no study was conducted between 2005 and 2013. Below is the list of the included studies:   1. *BAŞARAN et al. (2014). Adherence to Home Exercise Program among Caregivers of Children with Cerebral Palsy* 2. *Alwhaibi et al. (2022). Factors Affecting Mothers’ Adherence to Home Exercise Programs Designed for Their Children with Cerebral Palsy* 3. *Lillo-Navarro et al. (2019). Factors Associated with Parents’ Adherence to Different Types of Exercises in Home Programs for Children with Disabilities* 4. *MEDINA-MIRAPEIX et al (2017). Predictors of parents’ adherence to home exercise programs for children with developmental disabilities, regarding both exercise frequency and duration: a survey design* 5. *Rone-Adams et al. (2004). Stress and Compliance with a Home Exercise Program Among Caregivers of Children with Disabilities* 6. *Sel et al., (2020). Parents of children with physical disabilities perceive that characteristics of home exercise programs and physiotherapists’ teaching styles influence adherence: a qualitative study* 7. *Tahayneh et al., (2020), Factors affecting mother’s adherence towards cerebral palsy home exercise program among children at hebron and bethlehem, Palestine* 8. *Gmmash et al. (2021). Parental Adherence to Home Activities in Early Intervention for Young Children with Delayed Motor Development.* 9. *Galil et al., (2001). Compliance with home rehabilitation therapy by parents of children with disabilities in Jews and Bedouin in Israel* 10. *Tétreault et al., (2003).  Home activity programs in families with children presenting with global developmental delays: evaluation and parental perceptions* 11. *Louka-Lazouri et al., (2020). Investigation of parents' compliance in physical therapy at home with toddlers diagnosed with cerebral palsy* 12. *Demeke et al., (2023). Home-based therapy and its determinants for children with cerebral palsy, exploration of parents’ and physiotherapists’ perspective, a qualitative study, Ethiopia* 13. *Lillo-Navarro et al., (2019). Parents of children with physical disabilities perceive that characteristics of home exercise programs and physiotherapists' teaching styles influence adherence: A qualitative study* |
|  |  | Line 203: Please provide more details regarding the term adherence and how it is defined. Is it related to the "percent of suggested exercise done," or are exercise sessions done in a specific period of time? Does adherence change on a weekly basis, or is it defined as a single metric for the whole program duration? Does the duration of the program differ across the studies? | Page 9, line 1848,  Table1 | **We appreciate the reviewer for the constructive comment.**  *The more details regarding the term adherence were initially provided in S1 table especially in its column titled “Concept (and measure) of Adherence to HEP.” Due to the variations in measuring adherence across studies, we included in this review how each included study measured adherence. We revealed that measurement approaches ranged from self-designed questionnaires with a few questions to comprehensive, validated questionnaires with up to 28 items. Many studies utilized a 5-point Likert scale to measure adherence, capturing a range from "never" to "always" engaging in prescribed exercises. Additionally, qualitative methods such as semi-structured interviews and focus group discussions were employed to gather deeper insights into adherence challenges. This inclusion of both quantitative and qualitative measures highlights the complexity of assessing adherence and the diverse nature of home exercise programs. This refined overview is presented in the manuscript to better articulate the multifaceted approach to adherence measurement in pediatric rehabilitation contexts.*  We are sorry that you didn’t you see this table, which was initially submitted as supplement file, but now we submit it inside the manuscript to ensure that you see it. |
|  |  | Lines 209-214: Given the issues related to the studies, how is it possible to have three studies with 9 stars (10 is the maximum grade)? | Page 6, line 1041 | We are grateful for the reviewer's insightful comment and welcome the opportunity to clarify our quality assessment process for the studies included in this review. For both qualitative and quantitative studies, we employed validated and standardized scales to rigorously evaluate study quality. This approach was meticulously detailed in the methods section to ensure the replicability of our study.  ***Quality assessment***  *For quantitative studies, we utilized the Newcastle-Ottawa Scale (NOS) adapted for cross-sectional studies. This decision was informed by the nature of the review studies, which were explicitly of this type.^[29]^ The NOS evaluates the methodological quality of studies based on three critical domains: Selection, comparability, and outcome. Stars are awarded in these categories according to the guidelines provided in the NOS coding handbook, with up to five stars might be given for selection, two for comparability, and three for outcome. The total number of stars a study receives serves as an indicator of its quality.*  *In assessing the qualitative studies, the Critical Appraisal Skills Programme Tool (CASP) for Qualitative Research. The tool encompasses 10 criteria aimed at appraising a study's methodological rigour and validity.^[30]^ We adopted a rating approach to gauge the relative quality of these studies ^[31]^. Those meeting at least eight criteria were deemed 'high quality', those fulfilling 5-7 criteria were considered 'moderate quality' if they met 5-7 of the criteria, and studies meeting four or fewer criteria were categorized as 'poor quality'.*  *Given these rigorous assessment tools and criteria, the achievement of a 9-star rating by three studies reflects their high methodological quality within the constraints of cross-sectional study design. It's important to note that while perfection is unattainable, with 10 stars being the maximum theoretical grade, a 9-star rating signifies a study that excels in its methodological rigor, providing strong, reliable evidence within the limitations inherent to its design and scope.* |
|  |  | Line 208: The phrase "one was awarded 8 stars" is written twice. |  | **We apologize for this error; it was corrected as follows:**  *In appraising the quantitative studies, two studies were awarded nine stars, one study was received eight stars, four were awarded seven stars, and the remaining four studies also awarded seven stars (Table 1).* |
|  |  | Line 219: The sentence requires rephrasing. | Page 8, line 1415 | **We appreciate the reviewer for spotting this error, we have corrected as follows:**  ***Moreover, response rates were generally low across the studies, with no insights into the characteristics of non-responders. This omission makes assessing the representative of the participants challenging.***^[26]^ *It is plausible that parents who were most adherent to HEP may have been more willing to participate in the research because they saw it as relevant and valuable. In contrast, non-adherent parents might have chosen not to discuss their experiences and abstain from the research.^[26]^* |
|  |  | In the section "Quality assessment of the included articles," more details related to the metrics or measures used in the studies (223-238) are needed. | Page 9, line 1848,  Table1 | **We appreciate the reviewer for the constructive comment.**  *The more details regarding the term adherence were initially provided in table 1especially in its column titled “Concept (and measure) of Adherence to HEP.” Due to the variations in measuring adherence across studies, we included in this review how each included study measured adherence. We revealed that measurement approaches ranged from self-designed questionnaires with a few questions to comprehensive, validated questionnaires with up to 28 items. Many studies utilized a 5-point Likert scale to* *measure adherence, capturing a range from "never" to "always" engaging in prescribed exercises. Additionally, qualitative methods such as semi-structured interviews and focus group discussions were employed to* *gather deeper insights into adherence challenges. This inclusion of both quantitative and qualitative measures highlights the complexity of assessing adherence and the diverse nature of home exercise programs. This refined overview is presented in the manuscript to better articulate the multifaceted approach to adherence measurement in pediatric rehabilitation contexts.*  We are sorry that you didn’t you see this table, which was initially submitted as supplement file, but now we submit it inside the manuscript to ensure that you see it. |
|  |  | Line 173: Please provide the number of the table. | Page 14, line 1785 | **We apologize for this error; it was corrected as follows:**  *The only study where this association was not significant ^[35]^ is characterized by a weak methodological design (Table* ***1****).* |
|  |  | Line 260: The name of the section is "Caregiver-related factors," but parental-related factors are presented. Please explain. | Whole document | We apologize for this error, it’s parental-related factors. We have corrected in whole document. |
|  |  | In the results section, a description of the exercise sessions across the studies is needed to understand whether there are differences across the session settings. Thus, besides child/parental/therapist-related factors, program-related factors may exist too. | Page 16, line 1892 | Thank you for your valuable comment. Upon reflection, we recognize that the distinction between program-related factors and the roles of therapists might not have been clearly articulated in our manuscript. Indeed, as the exercise programs are designed and often tailored by physiotherapists, the characteristics of these programs, including session settings, inherently reflect therapist-related decisions and inputs. In our systematic review, when categorizing factors influencing home exercise program adherence (HEPA) among parents of children with cerebral palsy (C-CP), we broadly classified these under child-related, caregiver-related, and therapist-related factors. The latter was intended to encapsulate not only the direct guidance and support provided by therapists but also their critical role in designing and adjusting the exercise programs. This includes determining the frequency, duration, specificity, and delivery mode of the exercises, which are pivotal program-related factors that can significantly impact adherence.  However, we realize that our manuscript may not have explicitly conveyed the nuanced way in which program-related factors are integrated into the broader category of therapist-related factors. To clarify this in our review, we will refine our discussion to explicitly state that program-related factors, as determined by physiotherapists, are considered a subcategory of therapist-related factors. This clarification will highlight our understanding that the design and implementation of the exercise program—encompassing session frequency, duration, specificity, and mode of delivery—are indeed crucial components shaped by the therapists and are fundamental to influencing HEPA.  **Therefore, the following subsection of discussion was rigorously improved accordingly:**  **Discussion**  *To our knowledge, this is the first systematic review that objectively assesses the factors influencing adherence to home exercise programs among among caregivers, specifically mothers of C-CP. The evaluation of thirteen studies revealed a predominantly high quality of evidence.* ***Our findings categorize the influencing factors HEPA into three main categories: child-related factors (such as gross motor function), parental-related factors (including education level, depression, and anxiety, stress, emotional exhaustion, high self-efficacy, high knowledge and ability, and low perception of the barriers, and social support), and therapist-related factors. Importantly, therapist-related factors extend beyond direct guidance and support to include the design and tailoring of exercise programs. This encompasses decisions regarding the exercise amount, type, and adjustments to avoid adverse effects, all of which are pivotal for enhancing adherence.*** |

| **N** | **Reviewer 2** | **Reviewer’s comments** | **Page and line on which addressed** | **How comments were addressed** |
| --- | --- | --- | --- | --- |
| 1 | **Title & Abstract** | Reviewer #2: The manuscript presents a comprehensive review of factors influencing adherence to home exercise programs (HEP) for children with cerebral palsy, as perceived by their parents. The authors systematically searched databases up to March 2023 and included both quantitative and qualitative studies in their analysis. The findings are segmented into three main categories: child-related factors, caregiver-related factors, and therapist's guidance and advice. The review highlights the multifaceted nature of adherence to HEP, including the influence of the child's gross motor function, the caregiver's education level, depression, anxiety, self-efficacy, and the quality of therapist support | Whole document | Thank you very much for the overall appreciation of the quality of this manuscript. |
| 2 |  | 1. The manuscript could be strengthened by including graphical representations of the findings to better visualize the relationships between various factors and adherence. Including plots and diagrams can make the manuscript more impactful and is strongly recommended. | Page 13, line 1753 | We sincerely appreciate the reviewer's insightful comments, which have guided us in refining our manuscript to visualize the relationships between various factors and adherence.  Following the reviewer's recommendation, we have included a hierarchical diagram titled "Factors influencing the adherence to home exercise programs among parents of children with cerebral palsy." This diagram visually organizes the factors influencing adherence into three primary categories: Parental related Factors, Child-Related Factors, and Therapist-Related Factors. Each category is further subdivided into specific subcategories. This graphical representation enhances the clarity and accessibility of our findings, allowing readers to quickly grasp the multifaceted nature of adherence determinants.  **Figure 2: Factors influencing the adherence to home exercise programs among parents of children with cerebral palsy.**    We have also included the following flowchart figure.  **Figure 1. Flow chart of study selection**   |
| 3 | **Material and Methods** | 2. The narrative synthesis approach is well justified given the heterogeneity of the studies included. However, the authors might consider a more detailed explanation of how they addressed potential biases within the studies analyzed and the impact of study quality on the overall findings.  The results section highlights areas of agreement and disagreement among the studies reviewed. The discussion section could be strengthened by a deeper analysis of why these discrepancies might exist, considering factors such as study design, population differences, and measurement tools used. |  | **We greatly appreciate the thoughtful and constructive comments provided by the reviewer**. **The authors have stringently improved the discussion section by adding the following paragraphs:**  *The deeper analysis into the discrepancies observed in the results section reveals that the diversity in study designs, population characteristics, and measurement tools used across the reviewed studies significantly influences these differences. Variability in methodologies for conceptualizing and measuring adherence—ranging from self-designed questionnaires in studies like Başaran et al^[35]^ and Rone-Adams et al^[25]^ to validated scales as used by Sel et al^[39]^—affects the comparability of reported adherence rates. Furthermore, the demographic characteristics of the study populations, such as parents' gender, age, and cultural background, contribute to varying adherence levels. For instance, the study by Alwhaibi et al^[23]^, which involved a predominantly female sample from Saudi Arabia, contrasts with Gmmash et al^[33]^, where a significant number of participants were female from the USA. This variance highlights how cultural factors and parental roles within different societies may influence adherence levels, as suggested by the high compliance among Bedouin parents in Galil et al^[40]^, indicating potential cultural differences in perceptions of disability and therapy.*  *The age range and developmental stage of children varied significantly across studies, from early infancy to late childhood. Studies like Medina-Mirapeix et al^[36]^ focused on younger children (0.5-6 years), potentially influencing adherence differently compared to studies involving older children, due to factors such as parental motivation and the child’s ability to participate actively in exercises. Notably, social support emerged as a critical factor in multiple studies, underscoring the importance of a supportive environment for enhancing adherence. The quality of the studies, varied, which could impact the reliability of the findings. Higher-quality studies like those rated with 9 stars on the quality measures provided more robust evidence compared to lower-rated studies. These elements collectively underscore the complex interplay between methodological approaches, population demographics, and cultural contexts in interpreting discrepancies among the studies reviewed, pointing to the necessity of a nuanced understanding of adherence within the specific context of home-based rehabilitation for children with disabilities.* |
|  |  | 5. Grammatical and typographical errors should be corrected to improve the readability of the manuscript. | Whole document | **We appreciate the reviewer for the constructive recommendation.**  *The manuscript was proofread by someone who is proficient in English as indicated in the manuscript with track changes.* |
