# Supplementary material for: The psychosocial determinants of adherence to home-based rehabilitation strategies in parents of children with cerebral palsy: A systematic review
Source: PLoS One. 2024 Jun 12;19(6):e0305432. doi: 10.1371/journal.pone.0305432 (PMC11168686; doi:10.1371/journal.pone.0305432)
Supplement: Attachment — Submitted filename: Rebuttal letter May 24, 2024.doc [file pone.0305432.s003.doc]

**Rebuttal letter**

We wish to express our sincere appreciation to the editor and reviewer for their insightful comments. We have done our best to address all the issues raised and we hope that the final version of this manuscript is much improved due to their comments.

**Note:** This table corresponds to the manuscript with track changes. It means that page numbers correspond to the manuscript with track changes.

| **N** | **Reviewer 1** | **Reviewer’s comments** | **Page and line on which addressed** | **How comments were addressed** |
| --- | --- | --- | --- | --- |
|  | **General comments** | 1. If the authors have adequately addressed your comments raised in a previous round of review and you feel that this manuscript is now acceptable for publication, you may indicate that here to bypass the “Comments to the Author” section, enter your conflict of interest statement in the “Confidential to Editor” section, and submit your "Accept" recommendation.  Reviewer #2: All comments have been addressed  Reviewer #3: All comments have been addressed  2. Is the manuscript technically sound, and do the data support the conclusions?  Reviewer #2: Yes  Reviewer #3: Yes  3. Has the statistical analysis been performed appropriately and rigorously?  Reviewer #2: Yes  Reviewer #3: Yes  4. Have the authors made all data underlying the findings in their manuscript fully available?  Reviewer #2: Yes  Reviewer #3: Yes  5. Is the manuscript presented in an intelligible fashion and written in standard English?  Reviewer #2: Yes  Reviewer #3: Yes  6. Review Comments to the Author  Please use the space provided to explain your answers to the questions above. You may also include additional comments for the author, including concerns about dual publication, research ethics, or publication ethics. (Please upload your review as an attachment if it exceeds 20,000 characters)  Reviewer #2: The author has revised the manuscript as per the reviewers recommendations. The manuscript can be accepted.  Reviewer #3: Thank you for submitting your work. | Whole document | **We really appreciate the reviewer 2 and reviewer 3 for the overall appreciation of the quality of this revised manuscript.** |
| 1 |  | Here are my comments on the manuscript:  1) Introduction: Overall, please check citation formatting. Sometimes, there is space in between citation and text. | Page 3-4, line 63-128 | **We appreciate the reviewer for the constructive comments:**  We have reviewed the Introduction section and ensured that all citation formatting is consistent. Any unnecessary spaces between citations and the text have been corrected. |
|  |  | 2) Material and methods: No comments. | NA | ***Thank you enormously for appreciation of the study methodology.*** |
|  |  | 3) Results: In table 1, there is no need to include title in first column, rather than that, I would suggest using the citation of particular paper. Also, there is too much text in table 1. Is it possible to divide table 1 into two tables or decrease the text in table 1. | Page 10, line 350. | **Thank you for your insightful comments. I have addressed your concerns in the following ways:**  **Removed Titles from the First Column:** Titles have been replaced with citations.  **Summarized Key Findings:** The key findings column has been summarized to reduce text while maintaining the essential information. |
|  |  | 4) Why are lines not numbered after line 251? | Page 6, Line 251 | ***We apologize for this error stemming from page break. We have corrected entire document.*** |
|  |  | 5) Title “Family Income and equipment” is not entirely in italics, is their reason for that. | Page 13, line 951 | ***We apologize for this error; entire title should be in italics as follow:***  ***Family Income and equipment*** |
|  |  | 6) Overall, please check your citations. In some of them, there is space and sometimes, there is not. | Whole document | **Thank you for the insightful comment.**  We have reviewed the entire document and ensured that all citation formatting is consistent. Any unnecessary spaces between citations and the text have been corrected. |
|  |  | 7) Please check for sentence errors in discussion. Line: “Therefore,, when planning treatments with families, therapists should consider their impact on adherence to these types of exercises”. Line: “It underscores the need to integrate “care “into the therapeutic equation, fostering a more holistic approach that considers the psychological, social, cultural of dimensions of rehabilitation”. | Page 16, line 1087 | **We apologize for this error; it was corrected as follows:**  Moreover, our findings suggest a nuanced consideration for the types of exercises prescribed, as caregivers may exhibit varying levels of adherence depending on the exercise's perceived complexity and benefits. These findings align with previous studies indicating that most parents perceive flexibility exercises as unpleasant and complicated activities.^[49]^ ***Therefore, when planning treatments with families, therapists should consider the impact of these types of exercises on adherence.*** |
